# Hemoglobin Himeji and inconsistent hemoglobin A1c values: a case report

**DOI:** 10.1186/s13256-017-1377-1

**Published:** 2017-07-26

**Authors:** Vânia Guedes, Rita Bettencourt-Silva, Joana Queirós, Maria da Luz Esteves, Maria José Teles, Davide Carvalho

**Affiliations:** 1 Family Medicine, Unidade de Saúde Familiar Faria Guimarães, Rua Faria Guimarães 915/931, 4200-292 Porto, Portugal; 2 Department of Endocrinology, Diabetes and Metabolism, Centro Hospitalar de São João E.P.E, Porto, Portugal; 3 Faculty of Medicine, Instituto de Investigação e Inovação em Saúde, University of Porto, Porto, Portugal; 4 Family Medicine, Unidade de Saúde Familiar Egas Moniz, Santa Maria da Feira, Portugal; 5 Department of Clinical Pathology, Centro Hospitalar de São João E.P.E, Porto, Portugal

**Keywords:** Diabetes mellitus, Hemoglobin A1c, Hemoglobinopathies, Mutations, Hemoglobin Himeji

## Abstract

**Background:**

Hemoglobin A1c is used to evaluate the glycemic control in patients with diabetes and is a risk marker for chronic complications of diabetes. Hemoglobin variants are reported to falsely lower or increase hemoglobin A1c test results. We present a case report of a patient with diabetes with discrepancy between fasting plasma glucose and hemoglobin A1c due to the presence of hemoglobin Himeji, a clinically silent and very rare hemoglobinopathy.

**Case presentation:**

A 76-year-old white woman, born and living in Portugal, with type 2 diabetes presented to the family physician for a routine visit. She had no active complaints, including history or symptoms of hypoglycemia, and her physical examination was unremarkable. A review of her laboratory data showed fasting plasma glucose of 190 mg/dL and a hemoglobin A1c of 4.1%. The remaining blood test results were clinically insignificant; a further review of her laboratory data over the past 4 years revealed that her fasting plasma glucose had ranged from 130 to 250 mg/dL and hemoglobin A1c was consistently lower than 5%. A study of hemoglobins detected 32.8% of abnormal hemoglobin. Genetic sequencing identified a heterozygous mutation compatible with hemoglobin Himeji (c.422C>A; p.Ala141Asp). We tracked her family (three sons, six grandchildren, and two greatgrandchildren) for the presence of this hemoglobin variant, but none had this hemoglobinopathy.

**Conclusions:**

Despite the advantages of hemoglobin A1c in the follow-up and treatment of diabetes, the factors that interfere with its results must be known to ensure a correct estimation of the degree of glycemic control and a proper management of the disease. Therefore, health professionals should suspect the existence of hemoglobin variants when: the hemoglobin A1c value is above 15% or below the lower limit of its reference interval; there is a significant modification in its result coinciding with a change in assay methods; and there is a low correlation between plasma glucose and hemoglobin A1c. In patients with hemoglobin Himeji, alternate ways of monitoring glycemic control (fructosamine or glycated serum albumin) should be used.

## Background

Glycated hemoglobin (Hb) A1c (HbA1c) is a term used to describe Hb that has been irreversibly linked to glucose through a nonenzymatic reaction [1, 2]. HbA1c testing is used to document the degree of glycemic control in patients with diabetes mellitus, because its value reflects the mean glycemia of the last 120 days, the erythrocyte lifespan average [3]. It is also useful to determine the risk for the development and progression of complications related to this chronic disease [1, 2].

Several assay methods, certified by the National Glycohemoglobin Standardization Program (NGSP) and calibrated to the Diabetes Control and Complications Trial (DCCT) reference, can be used to determine HbA1c value [1, 2, 4]. However, several factors can interfere with various methods, affecting the accuracy of their measurements [1, 2, 5, 6]. Hb variants are reported to falsely lower or increase HbA1c test results [7, 8]. We present a case report of a patient with diabetes with discrepancy between fasting plasma glucose (FPG) and HbA1c, due to the presence of Hb Himeji, a clinically silent and very rare hemoglobinopathy.

## Case presentation

A 76-year-old white woman born and living in Portugal had a medical history of type 2 diabetes mellitus (without known microvascular or macrovascular complications) for more than 15 years, as well as hypertension, hyperlipidemia, and depression. She was treated with metformin (1500 mg per day), losartan/hydrochlorothiazide (100/25 mg per day), simvastatin (20 mg per day), aspirin (100 mg per day), and trazodone (150 mg per day).

She presented to the family physician for a routine visit. She had no active complaints, including history or symptoms of hypoglycemia. At the time of the evaluation, her physical examination was unremarkable, including her body mass index (24.3 kg/m^2^). A review of her laboratory data showed a FPG of 190 mg/dL and an HbA1c of 4.1%, which was measured by high-performance liquid chromatography (HPLC). The remaining blood test results were clinically irrelevant, including complete blood count (Table 1), lipid profile, liver and kidney functions, and iron metabolism. Inconsistent results were confirmed by analytical reassessment of HbA1c (4.5%, by HPLC) and FPG (236 mg/dL). Further review of her laboratory data over the past 4 years revealed that FPG had ranged from 130 to 250 mg/dL and HbA1c was consistently lower than 5%.

**Table 1:** Blood count

	Results	Reference values
Hemoglobin (g/dL)	15.0	12.0–16.0
Erythrocytes (×10^12^/L)	4.90	4.0–5.0
Hematocrit (%)	44.2	37–49
Reticulocyte count		
Percentage (%)	2.1	0.2–2.0
Absolute value (×10^9^/L)	105.6	50.0–100.0
Low fluorescence reticulocyte (%)	90.6	
Medium fluorescence reticulocyte (%)	8.6	
High fluorescence reticulocyte (%)	0.8	

**Fig. 1:**
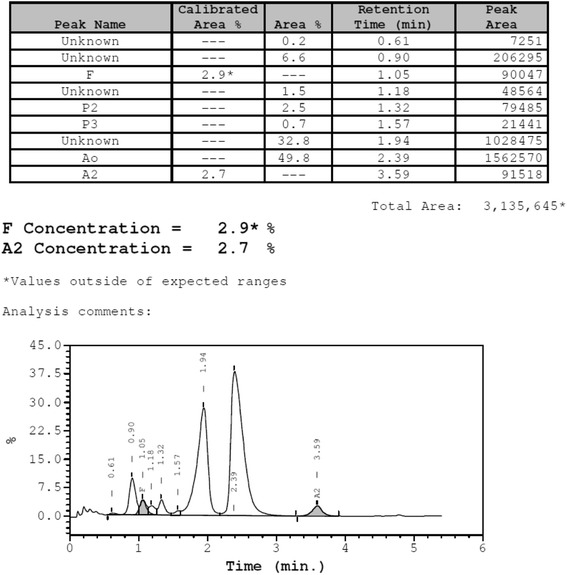
High-performance liquid chromatography analysis with Bio-Rad variant II – beta-thal short program. An abnormal peak of 32.8% was detected, with an earlier retention time than for A0

Given this discrepancy between HbA1c and plasma glucose, we hypothesized that she had an abnormal Hb, after excluding other potential factors such as anemia, hypertriglyceridemia, uremia, and chronic alcoholism [2]. Therefore, Hb variants were studied after obtaining informed consent. Hb electrophoresis showed an abnormal peak (32.8%) with an earlier retention time than for A0, suggesting the presence of an Hb variant (Fig. 1). The genetic sequencing of the beta-globulin gene revealed heterozygosity for the mutation c.422C>A (p.Ala141Asp) (p.Ala140Asp on the old nomenclature), corresponding to Hb Himeji.

Despite her denial of a relevant family history, namely of hematological diseases, we tracked her family (three sons, six grandchildren, and two greatgrandchildren) for the presence of this Hb variant, after obtaining informed consent. The study of Hb by HPLC was normal in all of them. Unfortunately, her two brothers were not available for study and her parents had died.

## Discussion

Currently more than 1200 Hb variants have been reported worldwide [9]. The most frequent are HbS and HbC and the majority arises from point mutations in the Hb chains [7]. Approximately 80% of hemoglobinopathies are asymptomatic, whereas the remaining 20% are associated with hemolytic anemia, polycythemia, or methemoglobinemia [7, 8, 10]. With the widespread determination of HbA1c, the identification of Hb variants that are clinically silent has increased in the last years [7, 10].

Hb variants interfere with several assay methods of HbA1c [2, 7]. Depending on the method, the same variant can produce unexpectedly high or low HbA1c values compared to glycemic control [2, 7]. Boronate affinity chromatography is generally considered to be less affected by abnormal Hbs [2]. Manual review of most cation-exchange chromatograms can also alert to the presence of aberrant peaks produced by a variant [2]. Furthermore, in the vast majority of patients heterozygous for an abnormal Hb, an appropriate method can be selected to accurately measure HbA1c [2]. However, if Hb variants affect the capacity of the Hb molecule to be glycated or if there are factors affecting erythrocyte turnover, the results will be inaccurate regardless of the Hb-based method used [2, 7].

Hb Himeji [9] was first described in 1986 in a Japanese male with diabetes mellitus [11] and subsequently in two Japanese families and in two members of a Portuguese family [10, 12, 13]. To the best of our knowledge, these are the only reports dealing with this pathologic condition. This abnormal Hb results from a mutation in heterozygosity characterized by the substitution of alanine for the aspartic acid at position 141 (140 in the old nomenclature) of the β chain of Hb [9]. Hb Himeji is a fast-moving Hb variant with an increased oxygen affinity, a mild molecular instability, and an increased glycation of the NH_2_ terminus of the β chain [11–13].

The HbA1c measurement may be falsely low or high depending on the assay method because of various mechanisms (differences in HPLC mobility, increased glycation, antigenic changes of the variant β chain, among others) [10]. We believe that the main factor for falsely low values of HbA1c in our patient was a reduced erythrocyte lifespan, with subsequent reticulocytosis. Electrospray ionization mass spectrometry (ESI-MS) is considered the most reliable method, but its excessive cost makes its use unlikely [7, 10]. Commercial assays that measure glycated serum proteins (fructosamine) or glycated serum albumin can accurately reflect glycemic control of patients with diabetes with Hb Himeji [2, 7, 14]. However, these assays translate the mean glycemia over a period of only 2 weeks and neither test has been correlated with the risk for chronic diabetes complications [1, 2, 7, 10]. Self-monitoring of blood glucose (SMBG) or continuous glucose monitoring also play an important role in assessing the efficacy of treatment and can be weighted in these cases [4].

Most patients with Hb Himeji are unaware of its presence, since they are asymptomatic [9]. However, they can have increased hemolysis and decreased erythrocyte survival. As such, in hematological stress situations, the compensatory reticulocytosis, observed in this patient, may be inadequate or absent and anemia can arise more easily and with greater severity than in normal situations [15]. In addition, HbA1c cannot be used to diagnose diabetes under conditions associated with increased red blood cell turnover [4]. To the best of our knowledge, Hb Himeji, by itself, is not associated with an increased risk of diabetes-related complications. However, insufficient treatment, which may result from the exclusive use of the HbA1c value for monitoring glycemic control, may lead to the development of these complications. The control of other cardiovascular risk factors, namely blood pressure, lipid profile, body weight, and tobacco smoking, as well as the adoption of a healthy lifestyle, may explain the absence of microvascular or macrovascular complications in this patient [16]. Since there are few cases of Hb Himeji in patients with diabetes, we do not know if these patients have any other protective factor for diabetic complications that could be expected after 15 years of poor diabetic control.

## Conclusions

Despite the advantages of HbA1c in the follow-up and treatment of diabetes, the factors that interfere with its results must be known, to ensure a correct estimation of the degree of glycemic control and a proper management of the disease. Therefore, health professionals should suspect the existence of Hb variants when: the HbA1c value is above 15% or below the lower limit of its reference interval; there is a significant modification in its result coinciding with a change in assay methods; and there is a low correlation between FPG/SMBG and HbA1c [2, 7, 8].

## Abbreviations

DCCT: Diabetes Control and Complications Trial
ESI-MS: Electrospray ionization mass spectrometry
FPG: Fasting plasma glucose
Hb: Hemoglobin
HbA1c: Hemoglobin A1c
HPLC: High-performance liquid chromatography
NGSP: National Glycohemoglobin Standardization Program
SMBG: Self-monitoring of blood glucose.

## Authors’ information

VG is currently a family medicine resident at Unidade de Saúde Familiar Faria Guimarães, Porto, Portugal. RBS is currently endocrinology resident at Centro Hospitalar de São João. MLE is an attending family medicine at Unidade de Saúde Familiar Egas Moniz, Santa Maria da Feira, Portugal. MJT is an attending clinical pathologist and JQ is an attending endocrinologist at Centro Hospitalar de São João. DC is currently the director of the endocrinology department at Centro Hospitalar de São João.
